# Clozapine Pharmacogenetic Studies in Schizophrenia: Efficacy and Agranulocytosis

**DOI:** 10.3389/fphar.2018.01049

**Published:** 2018-09-26

**Authors:** Shusuke Numata, Hidehiro Umehara, Tetsuro Ohmori, Ryota Hashimoto

**Affiliations:** ^1^Department of Psychiatry, Institute of Biomedical Science, Tokushima University Graduate School, Tokushima, Japan; ^2^Department of Psychiatry, Osaka University Graduate School of Medicine, Osaka, Japan; ^3^Molecular Research Center for Children's Mental Development, United Graduate School of Child Development, Osaka University, Osaka, Japan; ^4^Department of Pathology of Mental Diseases, National Institute of Mental Health, National Center of Neurology and Psychiatry, Tokyo, Japan

**Keywords:** clozapine, schizophrenia, clinical response, agranulocytosis, pharmacogenetics, SNP, GWAS, review

## Abstract

Clozapine is an efficacious atypical antipsychotic for treatment-refractory schizophrenia. Clinical response and appearance of adverse events vary among individual patients receiving clozapine, with genetic and non-genetic factors potentially contributing to individual variabilities. Pharmacogenetic studies investigate associations between genetic variants and drug efficacy and toxicity. To date, most pharmacogenetic studies of clozapine have been conducted through candidate gene approaches. A recent advance in technology made it possible to perform comprehensive genetic mapping underlying clinical phenotypes and outcomes, which allow novel findings beyond biological hypotheses based on current knowledge. In this paper, we will summarize the studies on clozapine pharmacogenetics that have extensively examined clinical response and agranulocytosis. While there is still limited evidence on clozapine efficacy, recent genome-wide studies provide further evidence of the involvement of the human leukocyte antigen (HLA) region in clozapine-induced agranulocytosis.

## Introduction

Approximately 30% of patients with schizophrenia are treatment-resistant (Meltzer, 1997). Clinical practice guidelines recommend clozapine in treatment-refractory schizophrenia (Warnez and Alessi-Severini, 2014), given that it has been shown to be superior for those resistant to treatment (Siskind et al., 2016). Clozapine may cause serious adverse events, such as agranulocytosis, cardiomyopathy, and myocarditis (Alvir et al., 1993; De Berardis et al., 2012; Alawami et al., 2014), so careful monitoring is needed during clozapine treatment. Clinical response and the presence of adverse events vary among individuals taking clozapine. Although the molecular mechanisms of clozapine action are still unclear (Yamamori et al., 2013, 2014; Hung et al., 2017; Kinoshita et al., 2017; Lee et al., 2017; Nakazawa et al., 2017), twin studies suggest that genetic factors may contribute to the variance in therapeutic and adverse effects of clozapine (Vojvoda et al., 1996; Horácek et al., 2001; Theisen et al., 2001; Wehmeier et al., 2005; Anil Yagcioglu et al., 2011).

Pharmacogenetic studies investigate the associations between genetic variants and drug efficacy and toxicity (Jorgensen and Williamson, 2008). To date, a number of pharmacogenetic studies of clozapine have been reported. In this paper, we summarize the studies on clozapine pharmacogenetics that have extensively examined clinical response and agranulocytosis.

### Clinical response to clozapine

Response rate for clozapine ranges from 32% in the short term to 39% in the long term among those with treatment-resistant schizophrenia (Siskind et al., 2017). Pharmacogenetic studies of clinical response to clozapine have focused on variations in the genes involved in the metabolism of clozapine [pharmacokinetics, e.g., the cytochrome P 450 (CYP) enzyme family] and the affinities of clozapine (pharmacodynamics, e.g., dopaminergic and serotonergic receptors). Positive candidate gene studies of the clinical response to clozapine are shown in Table 1.

**Table 1:** Positive findings of pharmacogenetic studies of clinical response to clozapine.

**Authors**	**Gene**	**Positive candidate genetic variants**	**Sample size**	**Ethnicity**	**CLZ dose**	**Treatment Duration**	**Clinical outcome**
**CYTOCHROME P450 (CYP) ENZYME RELATED GENES**							
Eap et al., 2004	*CYP1A2*	*1F	33 (4/29)	Four patients who were non-responder to clozapine in Lausanne, Konigsfelden, and Essen	Four non-responder patients: dose range 450–800 mg/day before augmentation treatment		CGI
Balibey et al., 2011	*CYP1A2*	*1F	97	Turkish	Mean dose: 308 ± 92 mg/day	18 weeks	BPRS
de Brito et al., 2015	*CYP1A2*	*1F	54	Brazilian	Mean dose of the non-responders: 593 ± 114 mg/day, mean dose of the responders: 535 ± 116 mg/da	About 2 years	BPRS
Piatkov et al., 2017	*CYP2C19*	*17	45	Australian (Caucasian, Asian, Pacific Islander, others)	No information	3 months and 12 months	No information
Lee et al., 2012	*ABCB1*	rs7787082, rs10248420	96	Korean	Mean dose: 319.0 ± 133.1 mg/day	More than 6 months	CGI
**DOPAMINE RELATED GENES**							
Potkin et al., 2003	*DRD1*	A-48G	15	Caucasian and African American	Mean dose: 460 ± 11 mg/day	5 weeks	BPRS
Hwang et al., 2007	*DRD1*	rs265976 in African-American samples	232	Caucasian and African American	No information	A minimum of 6 months	BPOS
Malhotra et al., 1999	*DRD2*	−141C Ins/Del	72	No information	No information	10 weeks	BPRS
Hwang et al., 2005	*DRD2*	Taq1A, Taq1B, rs1125394 in African-American samples	232	Caucasian and African American	No information	A minimum of 6 months	BPRS
Hwang et al., 2006	*DRD2*	Taq1B, rs1125394 in African-American samples	132	Caucasian and African American	No information	A minimum of 6 months	BPRS, BPOS
Huang et al., 2016	*DRD2*	rs2514218 in Caucasian subsamples	208	Caucasian and African American	No information	6 months	BPRS
Shaikh et al., 1996	*DRD3*	Ser9Gly (rs6280)	133	Caucasian	Dose range: 150–900 mg/day	At least 3 months on a stable regime of clozapine	GAS
Scharfetter et al., 1999	*DRD3*	Ser9Gly (rs6280)	32	Pakistani	Maximum dose: 600 mg/day	6 months	BPRS
Hwang et al., 2010	*DRD3*	rs2134655 in Caucasian samples, rs1394016 in African-American samples	232	Caucasian and African American	No information	A minimum of 6 months	BPOS, BNEG
Zhao et al., 2005	*DRD4*	48 bp variant number tandem repeat	81	Chinese	Dose range: 200–450 mg/day	At least 2 months treatment after clinical stabilization	PANSS
Hwang et al., 2012	*DRD4*	120-bp tandem repeat and 142bp/140bp in African American samples, 48 bp repeat in Caucasian samples	232	Caucasian and African American	No information	6 months	BPRS, BPOS
Woodward et al., 2007	*COMT*	Val108/158Met	86	Caucasian and African American	No information	6 weeks and 6 months	CIGT, COWAT, DSST
Xu et al., 2010	*SLC6A3*	rs2975226	160	Chinese	Mean dose: 415 ± 97 mg/day	At least 8 weeks	BPRS
**SEROTONIN RELATED GENES**							
Arranz et al., 1995a	*HTR2A*	T102C (rs6313)	149	Caucasian	Dose range: 125–600 mg/day	Stable for at least 3 months after clinical stabilization	GAS
Arranz et al., 1996	*HTR2A*	His452Tyr (rs6314)	153	Caucasian	Dose range: 125–600 mg/day	No information	GAS
Masellis et al., 1998	*HTR2A*	His452Tyr (rs6314)	185	Caucasian, African American, and Asian	No information	A minimum of 6 months	BPRS, CGI
Arranz et al., 1998b	*HTR2A*	G-1438A (rs6311)	274	Caucasian	Dose range: 125–600 mg/day	At least 3 months	GAS
Yu et al., 2001	*HTR2A*	T102C (rs6313)	99	Chinese	No information	No information	ERPs to auditory stimuli
Sodhi et al., 1995	*HTR2C*	Cys23Ser (rs6318)	162	Caucasian	Dose range: 125–600 mg/day	Stable for at least 3 months following clinical optimization	GAS
Yu et al., 1999	*HTR6*	C267T	99	Chinese	Mean dose: 271.6 mg/day for 267C/C, 287.5 mg/day for 267C/T, and 241.7 mg/day for 267T/T	At least 8 weeks	BPRS
Souza et al., 2010	*HTR3A*	rs1062613	140	Caucasian and African American	No information	A minimum of 6 months	BPRS
Rajkumar et al., 2012	*HTR3A*	rs1062613, rs2276302	101	Indian	Mean dose: 304.84 ± 119.04 mg/day	Stable dose regiments of clozapine for at least 12 weeks	BPRS
Kohlrausch et al., 2010	*SLC6A4*	HTTLPR/rs25531	116	Brazilian (European ancestry)	Mean dose: 540.91 mg/day	The same dose of clozapine at least 3 months	BPRS
**GENE-GENE INTERACTIONS**							
Arranz et al., 2000b	*HTR2A, HTR2C, SLC6A4*	His452Tyr (HTR2A), G-1438A (HTR2A), T102C (HTR2A),−330-GT/-244-CT (HTR2C), 5-HTTLPR (SLC6A4)	200	Caucasian	No information	No information	GAS
Hwang et al., 2011	*DRD1, DRD3*	rs686 (DRD1)-Ser9Gly (DRD3) in a Caucasian sample	232	Caucasian and African American	No information	A minimum of 6 months	BPRS
Bosia et al., 2015	*COMT, HTR1A*	Val158Met (rs4680) (COMT),−1019C/G (rs6295) (HTR1A)	107	Italian	The dosage was titrated up to 250mg/day and further augmentations or reductions were made on the basis of clinical response and plasma levels.	8 weeks and 16 weeks	PANSS
Rajagopal et al., 2018	*DRD4, COMT*	120-bp deplication (DRD4)-Val158Met (COMT)	93	Indian	No information	At least 12 weeks on a stable doses	BPRS

*CGI, Clinical Global Impression; BPRS, Brief Psychiatric Rating Scale; BPOS, positive symptom subscale (4 items of BPRS); BNEG, negative symptom subscale (3 items of BPRS); GAS, Global Assessment Scale; PANSS, Positive and Negative Symptom Scale; CIGT, Category Instance Generation Test; COWAT, Controlled Oral Word Association Test; DSST, Digit Symbol Subtest; ERP, event-related potential*.

#### Candidate gene approach

##### CYP enzyme related genes

Clozapine is metabolized primarily by *CYP1A2*, with additional contributions from *CYP2C19, CYP2D6*, and *CYP3A4* (Urichuk et al., 2008). To date, genetic variants in the *CYP1A2, CYP2C19, CYP2D6*, and *CYP3A4* genes as well as in the *CYP3A5, CYP3A7, CYP3A43*, and ATP binding cassette subfamily B member 1 (*ABCB1*) genes have been investigated in clozapine response studies (Arranz et al., 1995b; Eap et al., 2004; Balibey et al., 2011; Lee et al., 2012; Rajkumar et al., 2013b; de Brito et al., 2015; Piatkov et al., 2017). Five studies found significant associations between the clozapine treatment response and genetic variants in the *CYP1A2, CYP2C19*, and *ABCB1* genes (Eap et al., 2004; Balibey et al., 2011; Lee et al., 2012; de Brito et al., 2015; Piatkov et al., 2017). Eap and colleagues suggested that the CYP1A2^*^1F/^*^1F genotype may be associated with low plasma levels of clozapine and a lack of response to clozapine (*N* = 4) (Eap et al., 2004). Balibey and colleagues demonstrated that a positive response rate of clozapine was significantly lower in patients carrying the CYP1A2^*^1F/^*^1F genotype compared to those with at least one wild type allele for *CYP1A2* (*N* = 97) (Balibey et al., 2011). De Brite and colleagues replicated these findings (*N* = 54) (de Brito et al., 2015). Piatkov and colleagues demonstrated that homozygote CYP2C19^*^17 carriers were five times more likely to exhibit improvement on clozapine treatment (*N* = 45) (Piatkov et al., 2017). Furthermore, Lee and colleagues demonstrated that rs7787082 G and rs10248420 A alleles in the *ABCB1* gene were more common in non-responders (*N* = 96) (Lee et al., 2012).

##### Dopamine related genes

Clozapine exhibits a relatively high affinity for the dopamine D4 receptor (*DRD4*) and relatively lower affinities for the dopamine D1 receptor (*DRD1*), the dopamine D2 receptor (*DRD2*), and the dopamine D3 receptor (*DRD3*) (Meltzer, 1994). The combination of relatively high D1, low D2, and very high 5-HT2 receptor occupancy rates is unique to clozapine (Mauri et al., 2014). To date, genetic variants in the *DRD1, DRD2, DRD3, DRD4, and* dopamine D5 receptor (*DRD5)* genes as well as in dopamine -related genes, Catechol-O-methyltransferase (*COMT*) and solute carrier family 6 member 3 (*SLC6A3*) have been investigated in clozapine response studies, with positive associations observed in the *DRD1, DRD2, DRD3, DRD4, COMT*, and *SLC6A3* genes.

With respect to the *DRD1* gene (Potkin et al., 2003; Hwang et al., 2007; Lee et al., 2012), two studies found significant associations between genetic variants and the clozapine response (Potkin et al., 2003; Hwang et al., 2007). Potkin and colleagues demonstrated that rs4532 2/2 carriers were more likely to have a positive clozapine response following 5 weeks of treatment, while rs4532 1/2 carriers demonstrated a diminished clozapine response (*N* = 15) (Potkin et al., 2003). Hwang and colleagues demonstrated that rs265976 AC carriers were more likely to be non-responders to clozapine with a minimum of 6 months treatment in an African American sample (*N* = 49). They also investigated an association between three-marker haplotype (rs265981, rs4532, and rs686) and clozapine response, demonstrating that the T-G-A haplotype was associated with poor response in a Caucasian sample (*N* = 183), while the T-G-G haplotype was associated with better response in an African American sample (*N* = 49) (Hwang et al., 2007). With respect to the *DRD2* gene (Arranz et al., 1998a; Malhotra et al., 1999; Hwang et al., 2005, 2006; Lee et al., 2012), three studies found significant associations between genetic variants in this gene and the clozapine response (Malhotra et al., 1999; Hwang et al., 2005, 2006). Malhotra and colleagues investigated an association between 141C Ins/Del (rs1799732) and clozapine response in a 10 week treatment, demonstrating that Del- subjects had a five-fold greater reduction in psychotic symptoms as compared to Del+ subjects (*N* = 72) (Malhotra et al., 1999). Additionally, Hwang and colleagues investigated an association between 12 single nucleotide polymorphisms (SNPs) in the *DRD2* gene and clozapine response for a minimum of 6 months treatment, identifying 3 SNPs (Taq1A, Taq1B, and rs1125394) only in an African-American sample (*N* = 49) and several haplotypes in both Caucasian (*N* = 183) and African-American samples (*N* = 49) as being predictive of a clozapine response (Hwang et al., 2005). This same group later investigated the effect of the same 12 SNPs in the *DRD2* gene on clozapine response evaluated by overall, positive, and negative symptoms in smaller sample set (*N* = 35) and demonstrated that 2 SNPs (Taq1B and rs1125394) were associated with overall and positive symptom response to clozapine in an African-American sample (Hwang et al., 2006). However, a recent meta-analysis (total *N* = 596) suggests no association between 141C Ins/Del and a clozapine treatment response (Gressier et al., 2016). With respect to the *DRD3* gene (Gaitonde et al., 1996; Shaikh et al., 1996; Malhotra et al., 1998; Scharfetter et al., 1999; Arranz et al., 2000b; Barlas et al., 2009; Hwang et al., 2010; Lee et al., 2012), three studies found significant associations between genetic variants and the clozapine response (Shaikh et al., 1996; Scharfetter et al., 1999; Hwang et al., 2010). Shaikh and colleagues investigated an association between Ser-9-Gly polymorphism (rs6280) in the *DRD3* gene and clozapine response for at least 3 months of treatment (*N* = 79), demonstrating that the genotype Ser-9/Ser-9 was more frequent in the non-responders than in responders (Shaikh et al., 1996). Scharfetter and colleagues replicated this finding in patients treated with clozapine for 6 months (*N* = 32) (Scharfetter et al., 1999). However, a recent meta-analysis (total *N* = 852) suggests no association between Ser-9-Gly and a clozapine treatment response (Gressier et al., 2016). Hwang and colleagues demonstrated an association of better clozapine response for a minimum of 6 months with the A allele of rs2134655 in a Caucasian sample (*N* = 183) and the T allele of rs1394016 in an African-American sample (*N* = 49) (Hwang et al., 2010). With respect to the *DRD4* gene (Shaikh et al., 1993, 1995; Kerwin et al., 1994; Rao et al., 1994; Rietschel et al., 1996; Kohn et al., 1997; Kaiser et al., 2000; Zhao et al., 2005; Hwang et al., 2012; Lee et al., 2012; Rajagopal et al., 2018), two studies found significant associations between genetic variants and the clozapine response (Zhao et al., 2005; Hwang et al., 2012). Zhao and colleagues investigated an association between the 48 bp variant number tandem repeat (VNTR) polymorphism in the *DRD4* gene and clozapine response for at least 2 months of treatment after clinical stabilization (*N* = 81), demonstrating that the frequencies of 5 allele and 5/5 genotype were higher among the non-responders than in responders (*N* = 81) (Zhao et al., 2005). Hwang and colleagues demonstrated an association between 120-bp 1-copy allele and intron I (G)n 142/140 bp genotype and poor clozapine responders for 6 months of treatment in an African American sample (*N* = 49), and an association between 48 bp repeat polymorphism and better clozapine response in a Caucasian sample (*N* = 183) (Hwang et al., 2012). With respect to the *DRD5* gene, there were no significant associations between genetic variants and the clozapine response (Hwang et al., 2012). With respect to the *COMT* gene (Woodward et al., 2007; Bosia et al., 2015; Rajagopal et al., 2018), two studies found a significant association between the *COMT* Val108/158Met polymorphism (rs4680) and the clozapine response (Woodward et al., 2007; Bosia et al., 2015). Woodward and colleagues investigated an association between this polymorphism and clozapine response for 6 weeks and 6 months of treatment (*N* = 86), demonstrating that both the Met/Met and Val/Met groups showed greater improvement in attention and verbal fluency domains compared to the Val/Val group (Woodward et al., 2007). Bosia and colleagues demonstrated a greater improvement in the Val/Val group compared to both the Val/Met and Met/Met groups in the negative symptom response for 8 and 16 weeks of treatment with clozapine (*N* = 107) (Bosia et al., 2015). With respect to the *SLC6A3* gene, Xu and colleagues demonstrated that the 71T allele of rs2975226 (T-71A) in the *SLC6A3* gene occurred more frequently in the responders than in non-responders following at least 8 weeks of treatment with clozapine (Xu et al., 2010).

##### Serotonin related genes

Clozapine has a high affinity for the 5-hydroxytryptamine receptor 2A (*HTR2A*), the 5-hydroxytryptamine receptor 2C (*HTR2C*), the 5-hydroxytryptamine receptor 6 (*HTR6*), and the 5-hydroxytryptamine receptor 7 (*HTR7*) (Meltzer, 1994). To date, genetic variants in the *HTR2A, HTR2C, HTR6*, and *HTR7* genes as well as in 5-hydroxytryptamine receptor 1A (*HTR1A*), 5-hydroxytryptamine receptor 3A (*HTR3A*), 5-hydroxytryptamine receptor 3B (*HTR3B*), 5-hydroxytryptamine receptor 5A (*HTR5A*), and solute carrier family 6 member 4 (*SLC6A4*) genes have been investigated in clozapine response studies, with positive associations observed in the *HTR2A, HTR2C, HTR6, HTR1A, HTR3A*, and *SLC6A4* genes.

With respect to the *HTR2A* gene (Arranz et al., 1995a, 1996, 1998b, 2000b; Masellis et al., 1995, 1998; Nöthen et al., 1995; Malhotra et al., 1996a; Lin et al., 1999; Schumacher et al., 2000; Yu et al., 2001; Lee et al., 2012), six studies found significant associations between genetic variants and the clozapine response (Arranz et al., 1995a, 1996, 1998b, 2000b; Masellis et al., 1998; Yu et al., 2001). Arransz and colleagues investigated an association between the T102C polymorphism (rs6313) in the *HTR2A* gene and clozapine response for at least 3 months of treatment following clinical stabilization (*N* = 149), demonstrating that homozygosity for the C102 allele was more frequent in the non-responders than in the responders (Arranz et al., 1995a). Yu and colleagues demonstrated an association between the 102C/C genotypes and higher N100 amplitude following clozapine treatment using event-related potentials to auditory stimuli (*N* = 98) (Yu et al., 2001). Consistent with these findings, a recent meta-analysis (total *N* = 868) suggests a significant association between the CC genotype and poor clozapine treatment response (Gressier et al., 2016). Arransz and colleagues also investigated an association between the his452tyr polymorphism (rs6314) in the *HTR2A* gene and clozapine response (*N* = 153), demonstrating that the Tyr452 allele occurred more frequently in the non-responders than in the responders (Arranz et al., 1996). Masellis and colleagues replicated this finding in subjects who received clozapine for a minimum of 6 month (*N* = 185) (Masellis et al., 1998). Consistent with these findings, a recent meta-analysis (total *N* = 671) suggests a significant association between C allele or C carriers or CC genotype and better clozapine treatment response compared to T allele or T carriers or TT genotype (Gressier et al., 2016). Arranz and colleagues also examined an association between the G-1438A polymorphism (rs6311) in the *HTR2A* gene and clozapine response (*N* = 274), demonstrating that homozygosity for the G-1438 allele was more frequent in the non-responders than the responders (Arranz et al., 1998b). However, a recent meta-analysis (total *N* = 547) suggests no association between the G-1438A polymorphism and clozapine treatment response (Gressier et al., 2016). With respect to the *HTR2C* gene (Sodhi et al., 1995; Malhotra et al., 1996b; Rietschel et al., 1997; Masellis et al., 1998; Arranz et al., 2000b; Schumacher et al., 2000), two studies found a significant association between genetic variants and the clozapine response (Sodhi et al., 1995; Arranz et al., 2000b). Sodhi and colleagues investigated an association between the Cys23Ser polymorphism (rs6318) in the *HTR2C* gene and clozapine response for at least 3 months treatment after clinical stabilization (*N* = 162), demonstrating that Ser allele carriers were more likely to show a response to clozapine (Sodhi et al., 1995). However, a recent meta-analysis suggests no association between the Cys23Ser polymorphism and clozapine treatment response in a Caucasian sample (Gressier et al., 2016). With respect to the *HTR6* gene (Yu et al., 1999; Masellis et al., 2001; Lee et al., 2012), one study found a significant association between a genetic variant and the clozapine response (Yu et al., 1999). Yu and colleagues examined the association between the C267T polymorphism in the *HTR6* gene and clozapine response for at least 8 weeks of treatment (*N* = 99), demonstrating that the 267T/T genotype was more frequent in responders than in non-responders (Yu et al., 1999). With respect to the *HTR7* gene, there was not a significant association between the pro279leu polymorphism and the clozapine response (Masellis et al., 2001). With respect to the *HTR1A* gene (Masellis et al., 2001; Bosia et al., 2015), one study found a significant association between a genetic variant and the clozapine response (Bosia et al., 2015). Bosia and colleagues examined the association between the −1019C/G (rs6295) polymorphism in the *HTR1A* gene and clozapine response for 8 and 16 weeks of treatment (*N* = 107), demonstrating a greater improvement in the G/G group compared to the C/C group (Bosia et al., 2015). With respect to the *HTR3A* gene (Arranz et al., 2000b; Gutiérrez et al., 2002; Souza et al., 2010; Lee et al., 2012; Rajkumar et al., 2012), *two* studies found significant associations between genetic variants and the clozapine response (Souza et al., 2010; Rajkumar et al., 2012). Souza and colleagues demonstrated the T allele of rs1062613 in the *HTR3A* gene was associated with a good clozapine treatment response when used for at least 6 months (*N* = 140) (Souza et al., 2010). Similarly, Rajkumar and colleagues investigated an association of rs1062613 and rs2276302 in the *HTR3A* gene with the clozapine response, demonstrating that the T allele of rs106263 and the G allele of rs2276302 were associated with a good clozapine treatment response when presented with a stable dose for at least 12 weeks (*N* = 101) (Rajkumar et al., 2012). Consistent with these findings, a recent meta-analysis suggested that there may be an association between the T allele of rs1062613 and an improved response to clozapine (Gressier et al., 2016). With respect to the *HTR3B and HTR5A* genes (Arranz et al., 2000b; Birkett et al., 2000; Gutiérrez et al., 2002; Souza et al., 2010), there were no significant associations between genetic variants and the clozapine response. With respect to the *SLC6A4* gene (Arranz et al., 2000a,b; Schumacher et al., 2000; Tsai et al., 2000; Kohlrausch et al., 2010), two studies found significant associations between genetic variants and the clozapine treatment response (Arranz et al., 2000b; Kohlrausch et al., 2010). Arranz and colleagues demonstrated a significant association between the biallelic polymorphism in the promoter region of the *SLC6A4* gene, 5-HTTLPR, and the clozapine response in a Caucasian sample (*N* = 200) (Arranz et al., 2000b), and they found a similar trend in a larger sample (*N* = 268) (*p* = 0.08) (Arranz et al., 2000a). Kohlrausch and colleagues demonstrated that the short allele of HTTLPR/rs25531 occurred more frequently in the non-responders than in responders (Kohlrausch et al., 2010) (*N* = 116).

##### Glutamatergic receptors

Accumulating evidence suggests the potential for clozapine to act on glutamatergic neurotransmission (Heresco-Levy, 2003). To date, genetic variants in the solute carrier family 1 member 2 (*SLC1A2*), solute carrier family 6 member 9 (*SLC6A9*), glutamate ionotropic receptor AMPA type subunit 1 (*GRIA1*), glutamate metabotropic receptor 2 (*GRM2*), and glutamate decarboxylase 1 (*GAD1*) genes, glutamate ionotropic receptor NMDA type subunit 1 (*GRIN1*), glutamate ionotropic receptor NMDA type subunit 2A (*GRIN2A*), glutamate ionotropic receptor NMDA type subunit 2B (*GRIN2B*), have all been examined in clozapine treatment response studies, with no significant associations observed following correction for multiple testing (Hong et al., 2001; Hwang et al., 2011; Taylor et al., 2016, 2017).

### Gene-Gene interactions

Several trials have been performed to investigate a gene-gene interaction between multiple candidate genes and the clinical response to clozapine. Arranz and colleagues examined an association between 19 genetic polymorphisms in nine clozapine-targeted receptor subtypes and a neurotransmitter transporter and the response to clozapine, demonstrating that the strongest association was with a combination of six polymorphisms (*HTR2A* T102C, *HTR2A* his452tyr, *HTR2C*−330-GT/-244-CT, *HTR2C* Cys23Ser, *SLC6A4* 5-HTTLPR, and *H2*−1018-G/A) (*N* = 200) (Arranz et al., 2000b). However, Schumacher et al. could not replicate this finding (Schumacher et al., 2000). Hwang and colleagues demonstrated the most straightforward gene-gene- interaction effect of *DRD1* rs686 and *DRD3* Ser9Gly on the clozapine treatment response when taken for a minimum of 6 months in a Caucasian sample (*N* = 183) (Hwang et al., 2011). Bosia and colleagues demonstrated an additive effect of *COMT* Val158Met and *HTR1A*−1019 C/G on the clozapine treatment response when taken for 8 and 16 weeks (*N* = 107) (Bosia et al., 2015). Similarly, Rajagopal and colleagues demonstrated a significant gene-gene- interaction effect of *DRD4* 120-bp duplication and *COMT* Val158Met on the clozapine response when taken for at least 12 weeks (*N* = 93) (Rajagopal et al., 2018). Furthermore, Xu and colleagues conducted multivariate interaction analysis using 77 SNPs of 25 genes and demonstrated that the combination of rs6269 in the *COMT* gene and rs3813929 in the *HTR2C* gene may work as predictor to improve the clinical antipsychotic response (risperidone, clozapine, quetiapine, and chlorpromazine) in a large sample set (*N* = 995) (Xu et al., 2016).

### Non-candidate gene approach

To date, the authors are unaware of any genome-wide association studies (GWAS) of clozapine efficacy that have been published. Recent pharmacogenetics GWAS suggest genetic overlap between antipsychotic response and susceptibility to schizophrenia (Ikeda et al., 2015; Ruderfer et al., 2016), and two genetic variants, identified by GWAS as schizophrenia risk loci (Cross-Disorder Group of the Psychiatric Genomics Consortium., 2013; Schizophrenia Working Group of the Psychiatric Genomics Consortium., 2014), have shown to be associated with a significant clinical response to clozapine (Brandl et al., 2016; Huang et al., 2016). Randl and colleagues demonstrated a significant association between rs2535629 in the inter-alpha-trypsin inhibitor heavy chain H3 (*ITIH3*) gene and an improvement of negative symptoms after 6 months of clozapine treatment (*N* = 105) (Brandl et al., 2016). Additionally, Huang and colleagues demonstrated a significant association between rs2514218, located upstream of the *DRD2* gene, and clozapine response for 6 months of treatment (*N* = 208) (Huang et al., 2016).

### Clozapine-induced agranulocytosis

The occurrence of clozapine-induced agranulocytosis (CIA) is 0.8% at 1 year of administration of clozapine with reduced incidence after the first 6 months of clozapine treatment (Alvir et al., 1993). A recent meta-analysis suggests that 3.8% of patients exposed to clozapine will develop mild neutropenia (with an absolute neutrophil count of < 1,500/μl; Myles et al., 2018). A number of genetic studies of CIA and clozapine-induced agranulocytosis/granulocytopenia (CIAG) using samples from patients with schizophrenia have been conducted (Lieberman et al., 1990; Claas et al., 1992; Corzo et al., 1995; Yunis et al., 1995; Theodoropoulou et al., 1997; Turbay et al., 1997; Amar et al., 1998; Valevski et al., 1998; Meged et al., 1999; Dettling et al., 2000, 2001a,b, 2007; Lahdelma et al., 2001; Ostrousky et al., 2003; Mosyagin et al., 2004, 2005; Athanasiou et al., 2011; Anil Yagcioglu et al., 2016; van der Weide et al., 2017), many focusing on genetic variants in the genes within the major histocompatibility complex (MHC) region. Positive candidate gene studies of CIA and CIAG are shown in Table 2.

**Table 2:** Positive findings of pharmacogenetic studies of clozapine-induced agranulocytosis.

**Authors**	**Candidate genes/genetic variants**	**Sample size**	**Ethnicity**	**Clinical outcome**	**Definition of Agranulocytosis/Granulocytopenia**
**HUMAN LEUKOCYTE ANTIGEN (HLA) GENES**					
Lieberman et al., 1990	*HLA-B38, HLA-B38-DR4-DQw3*	6 cases and 25 controls	Mainly Jewish ancestry	CIA	A total white blood cell count of <3 × 10^9^/L and an absolute polymorphonuclear leukocyte count of <0.5 × 10^9^/L
Yunis et al., 1995	HLA-B38, HLA-DR4, HLA-B38-DR4-DQw3, HLA-DRB1*0402, HLA-DQB1*0302, HLA-DQA1*0301, HLA-DQB1*0301, HLA-DRB1*11, DRB1*0402-DRB4*0101-DQB1*0302, DQA1*0301 in Jewish patients, HLA-B7, HLA-DR2, HLA-DR2-DQw1, HLA-B7-DR2-DQw1, HLA-DQB1*0502, HLA-DQA1*0102, DRB1*1601-DRB5*02-DQB1*0502-OQA1*0102 in non-Jewish patients	31 cases and 52 controls	White European Jerish and non-Jerish	CIA	Absolute neutrophil count <500/mm^3^
Corzo et al., 1995	*HSP-70*	32 cases and 43 controls	Jerish and non-Jerish	CIA	No information
Turbay et al., 1997	*TNF*	33 cases and 33 controls	White European Jerish and non-Jerish	CIA	Absolute neutrophil count <500/μL
Amar et al., 1998	HLA-DQB1*0201	5 cases and 13 controls	Jerish and non-Jerish	CIAG	Agranulocytosis: total white blood cell count <3,000 mm^3^ and absolute polymorphonuclear count <500 mm^3^ Granulocytopenia: total white blood cell count <3,500 mm^3^ and absolute polymorphonuclear count <1,000 mm^3^
Valevski et al., 1998	*HLA-B38*	11 cases and 50 controls	Israeli Jerish	CIA	A granulocyte count of <500 mm^−3^
Dettling et al., 2001b	HLA-Cw*7, HLA-DQB*0502, HLA-DRB1*0101, HLA-DRB3*0202	31 cases and 77 controls	Non-Jewish German Caucasian	CIA	Absolute neutrophil count of <500 per mm^3^
Dettling et al., 2001a	HLA-DQB*0502, HLA-DRB5*02	30 cases and 77 controls	Non-Jewish German Caucasian	CIA	Absolute neutrophil count of <500 × 10^9^/L
Dettling et al., 2007	HLA-DRB5*0201, HLA-Cw7-B18, HLA-Cw7-B39, HLA-DRB5*0201-DRB4*000, HLA-Cw7-B18-DRB5*000, HLA-Cw7-B39-DRB5*000, HLA-Cw7-B44-DRB5*000	42 cases and 75 controls	Non-Jewish German Caucasian	CIA	Absolute neutrophil count of <500 per mm^3^
Lahdelma et al., 2001	*HLA-A1, HLA-A28, HLA-B16*	22 cases and 19 controls or 120 controls	Finnish	CIAG	Agranulocytosis: neutrophil granulocytes <0.5 × 10^9^/L Granulocytopenia: neutrophil granulocytes <1.5 × 10^9^/L
**NON-HUMAN LEUKOCYTE ANTIGEN (HLA) GENES**					
Athanasiou et al., 2011	*HLA-DQB1*	Cohort I:33 cases and 54 controls Cohort II:49 cases and 78 controls	Cohort I:United States, Russia, and South Africa Cohort II:non-Jewish German Caucasian	CIA	absolute neutrophil count * ≤* 500 cells/mm^3^
Ostrousky et al., 2003	*NQO2*	18 cases and 80 controls	Jerish	CIA	A neutrophil count <500 cells/ul
Mosyagin et al., 2004	*MPO*	49 cases and 78 controls	German White	CIA	Absolute neutrophil count of <500 per mm^3^
Athanasiou et al., 2011	*DRD1, CSF2RB, NTSR1*	33 cases and 54 controls	United States, Russia, and South Africa	CIA	Absolute neutrophil count * ≤* 500 cells/mm^3^
van der Weide et al., 2017	*ABCB* C3435T (rs1045642) and *NQO2* G1541A for CIA, *ABCB1* C3435T, *GSTT1*, and *GSTM1* for neutropenia	69 cases and 241 controls	Dutch	CIA, neutropenia	CIA: at least one neutrophil count * ≤* 500 ul^−1^ Neutropenia: at least one neutrophil count between 500 ul^−1^ and 1500 ul^−1^ or two neutrophil counts <2,000 ul^−1^

*CIA, Clozapine-Induced Agranulocytosis; CIAG, Clozapine-induced agranulocytosis/Clozapine-induced granulocytopenia*.

#### Candidate gene approach

##### Human leukocyte antigen (HLA) genes

In patients developing CIA (*N* = 6) compared with controls (*N* = 25), Lieberman and colleagues demonstrated that the occurrence of *HLA-B38* as well as the haplotype of *HLA-B38, DR4*, and *DQw3*, was more frequent (Lieberman et al., 1990). The same laboratory subsequently analyzed them separately by Ashkenazi Jewish patients and non-Jewish patients, conforming their previous finding in the Ashkenazi Jewish samples and demonstrating a significant association of *HLA-B7* and *DR2* with CIA in non-Jewish patients (Yunis et al., 1995). They also demonstrated that the variants in the heat-shock protein 70 (*HSP-70*) and tumor necrosis factor (*TNF*) genes were associated with CIA (Corzo et al., 1995; Turbay et al., 1997). Additionally, Amar and colleagues demonstrated that HLA-DQB1^*^020 significantly occurred more frequently in subjects who developed CIAG compared to those who did not develop those complications (5 CIAG cases vs. 13 controls) (Amar et al., 1998). Valevski and colleagues replicated the involvement of *HLA-B38* in CIA in Jewish Israeli samples (11 CIA cases vs. 50 controls) (Valevski et al., 1998). Dettling and colleagues demonstrated a significant association of HLA-Cw^*^7, DQB^*^0502, DRB1^*^0101, and DRB3^*^0202 with CIA in non-Jewish Caucasian samples (31 CIA cases vs. 77 controls) (Dettling et al., 2001b). The same laboratory also demonstrated a significantly higher frequency of HLA-DRB5^*^02 and HLA-DQB^*^0502 in patients who developed CIA (Dettling et al., 2001a). Later, the same laboratory performed an association study of CIA by combining the HLA class I and class II genetic variants, demonstrating that HLA-DRDB5^*^0201 and several haplotypes of the HLA genetic variants were associated with CIA (Dettling et al., 2007). Furthermore, Lahdelma and colleagues demonstrated that the *HLA-B16* allele occurred more frequently in patients who developed CIAG (22 CIAG cases vs. 120 healthy controls) (Lahdelma et al., 2001), while Athanasiou and colleagues demonstrated that the *HLA-DQB1* “REC 21G” occurred more frequently in patients who developed CIA compared with controls in two independent cohorts (Athanasiou et al., 2011).

##### Non-human leukocyte antigen (HLA) genes

Ostrousky and colleagues demonstrated that four polymorphisms in the dihydronicotinamide riboside quinone oxidoreductase 2 (*NQO2*) gene were associated with CIA (18 CIA cases vs. 80 controls) (Ostrousky et al., 2003). Additionally, Mosyagin and colleagues demonstrated that myeloperoxidase (*MPO*)−463AA carriers occurred more frequently in patients who developed CIA compared with AG and GG-carriers combined (49 CIA cases vs. 78 controls) (Mosyagin et al., 2004). Furthermore, Athanasiou and colleagues showed that *DRD1*, neurotensin receptor 1 (*NTSR1*), and β chain of colony-stimulating factor 2 receptor (*CSF2RB*) as well as *HLA-DQB1* and *HLA-C* were associated with CIA (33 CIA cases vs. 54 controls) (Athanasiou et al., 2011). Van der Weide and colleagues demonstrated that *NQO2* 154AA and *ABC1* 3435TT occurred more frequently in patients who developed CIA compared with controls (31 CIA cases vs. 241 controls) (van der Weide et al., 2017). They also showed that for patients who developed neutropenia (*N* = 38), compared to controls (*N* = 241), *ABCB1* 3435TT and homozygosity for glutathione S-transferase theta 1 (*GSTT1*)null occurred more frequently, but glutathione S-transferase mu 1 (*GSTM1*)null occurred less frequently (van der Weide et al., 2017).

#### Non-candidate gene approach

Tiwari and colleagues conducted exome sequence analysis and did not find any genetic variants associated with CIA in Finnish patients after Bonferroni correction (13 CIA cases and 11 cases with severe neutropenia vs. 26 controls) (Tiwari et al., 2014). Meanwhile, Goldstein and colleagues conducted GWAS, whole-exome sequencing, and HLA allele imputation and were able to show that *HLA-B* 158T and *HLA-DQB1* 126Q were associated with CIAG (Goldstein et al., 2014). Legge and colleagues utilized GWAS, imputed HLA alleles, exome array, and copy-number variation analyses in a European population and subsequently combined the data of Goldstein et al. (2014) and Legge et al. (2017). In their meta-analysis of GWAS, they demonstrated that rs149104283, an intronic transcript of *SLCO1B3* and *SLCO1B7*, was associated with clozapine-induced neutropenia. The authors of this paper conducted GWAS of CIAG in Japanese samples (50 CIAG cases vs. 2,905 controls) and identified rs1800625 in the HLA region as the CIAG candidate locus (Saito et al., 2016). A classical HLA analysis was subsequently conducted, demonstrating a significant association of HLA-B^*^59:01 with CIAG. However, we failed to replicate the risk SNPs on clozapine-associated neutropenia previously identified by Legge et al. (2017) in a Japanese population (Saito et al., 2017).

## Discussion

In the present article, we summarize clozapine pharmacogenetic studies, separated by clinical response and CIA. To date, pharmacogenetic studies of clozapine in schizophrenia have been mostly conducted through candidate gene approaches. Genetic variants in the CYP enzyme family, dopamine, and serotonin receptor genes have been extensively examined as pharmacokinetic and pharmacodynamics candidates related to clozapine efficacy. Although there is limited evidence, the most recent meta-analyses indicate that only three SNPs (rs6313 and rs6314 in the *HTR2A* gene and rs1062613 in the *HT3A* gene) are associated with a significant clozapine response (Gressier et al., 2016). Genetic variants in the genes within the MHC region have been extensively examined with respect to CIA. The results of these candidate studies indicate the HLA variants are implicated in developing CIA. A comprehensive screening of genetic variants linked to a significant clinical response to clozapine and CIA is critical because the selection of candidate genes is restricted to current knowledge about underlying biological mechanisms (Adkins et al., 2011). Recently, the field of clozapine pharmacogenetics has shifted from a candidate gene approach to a genome-wide approach. A genome-wide approach has successfully identified novel genes that are associated with CIA and provided further evidence of the involvement of the HLA variants in CIA (Goldstein et al., 2014; Saito et al., 2016). On the other hand, there are no known GWAS looking at the clinical response to clozapine, although there are several GWAS looking at the antipsychotic treatment response in patients with schizophrenia (McClay et al., 2011; Drögemöller et al., 2016; Ruderfer et al., 2016; Li et al., 2017; Yu et al., 2018). Yu and colleagues conducted GWAS in a large cohort (*n* = 3,792) and detected five loci (*CNTNAP5, MEGF10, PCDH7, SLC1A1*, and *TNIK*) associated with general antipsychotic treatment response (Yu et al., 2018). Interestingly, these loci did not include *DRD2*, although D2 receptor blockade in the brain is a general pharmacodynamic property of antipsychotics (Mauri et al., 2014).

Pharmacogenetic testing has the potential to help improve patient outcome, lower healthcare costs, and increase patient medication adherence (Gardner et al., 2014). Pharmacogenetic testing in psychiatry is not yet a standard of practice, however, its utilization is steadily increasing (Eum et al., 2016; Fabbri et al., 2018). Several studies have, indeed, assessed the clinical utility for risk genetic variants of CIA. The sensitivity and specificity of the *HLA-DQB1* 6672G.>C polymorphism for CIA in patients treated with clozapine, identified though candidate approach, was 21.5 and 98.4%, respectively (Athanasiou et al., 2011). The sensitivity and specificity of the HLA-B^*^59:01 for CIA, identified by GWAS, was 31.8 and 95.3%, respectively (Saito et al., 2016) and the sensitivity and specificity of *HLA-B* 158T and *HLA-DQB1* 126Q polymorphisms for CIA, identified by GWAS and whole-exome sequencing (Goldstein et al., 2014), was 41 and 85%, respectively (Girardin et al., 2018). Clinical application guidelines require HLA allele testing for CIA to have a sensitivity of ~50% (Girardin et al., 2018), therefore none of these have reached an acceptable threshold for clinical application. Conversely, we examined the diagnostic performance of non-risk allele (alleles except for HLA-B^*^59:01) on non-CIA among CIG patients and demonstrated a moderate, positive predictive value for detecting non-CIA subjects in the CIG group without the risk allele, suggesting a potential candidate for re-challenging with clozapine treatment in a Japanese population (Saito et al., 2016). Based on this finding, a re-challenging with clozapine following neutropenia in a patient with a low risk of CIAG (HLA-B^*^52:01/52:01) was successfully conducted (Yamaki et al., 2017). The decision regarding clozapine re-challenge or withdrawal in case of CIAG should be based on careful consideration of risk factors, which can be facilitated by genetic testing in the future (Wicinski and Weclewicz, 2018). Further efforts to identify strong and reproducible genetic variants related to the clinical response to clozapine and CIA are needed to develop appropriate pharmacogenetic testing of clozapine.

The major issue of pharmacogenetics studies is inconsistent findings among studies. The discrepancies between these studies might be caused by statistical issues (i.e., sample size, multiple testing) and methodological issues (i.e., study design, phenotype definition, genetic polymorphism, population stratification) (Ross et al., 2012). Indeed, each sample size of the clozapine pharmacogenetic studies was relatively small. Large samples are needed to have enough statistical power to detect the effects of genetic variants on clinical outcomes by creating a clozapine consortium (Saito et al., 2016, 2017) or performing meta-analyses (Gressier et al., 2016; Legge et al., 2017). A sample size of more than 900 participants will be needed in a pharmacogenetic study if a common variant is anticipated with a large effect (Ross et al., 2012). Additionally, most of the clozapine pharmacogenetic studies did not adjust for the multiple statistical comparisons, resulting in type I error. To adjust for multiple testing, a false discovery rate (FDR) correction will be useful. Furthermore, selection bias and information bias are confounding factors in both prospective cohort and case-control studies (Ross et al., 2012). Clinical responses to clozapine have been determined by several different evaluation scales, including the Clinical Global Impressions Improvement (CGI-I) score, the global assessment scale (GAS), the Brief Psychiatric Rating Scale (BPRS), and Positive and Negative Symptom Scale (PANSS). Linkage disequilibrium (LD) varies among ethnic population, which may affect cross-subpopulation comparisons when causal SNPs are not directly genotyped but rather captures by proxy SNPs (Ross et al., 2012). Population stratification occurs when ethnic subpopulations within the entire study population differ in terms of genotype frequency and risk of disease (Thomas and Witte, 2002). In addition, clozapine doses and treatment length as well as types of antipsychotics preceding clozapine administration differ among studies. Although the clozapine treatment length in clinical response studies ranges from 5 weeks to more than 6 months, Lieberman and colleagues reported that 76% of patients responded to clozapine administered up to 52 weeks and slow clozapine responders experienced 70% of their total improvement after 12 weeks of treatment with clozapine (Lieberman et al., 1994). Standardized treatment protocols and evaluations of clinical outcomes will be needed. Furthermore, both non-genetic and genetic factors play an important role in the clinical outcome. For example, age at onset, gender, severity of the illness, negative symptoms, extrapyramidal side effects, clozapine concentration, history of catatonia, smoking, hyper-somnolence, and cognitive deficits have all been associated with the clozapine treatment response (Perry et al., 1991; Lieberman et al., 1994; Miller et al., 1994; Umbricht et al., 2002; Semiz et al., 2007; Rajkumar et al., 2013a). To take these factors in analyses will be needed.

In conclusion, a number of clozapine pharmacogenetic studies have been performed based on candidate gene approaches. However, there is heterogeneity across studies and their results have been inconsistent. Reproducible genetic variants with large effect related to the clinical response to clozapine and CIA have not been detected so far. This field is beginning to shift from candidate gene approaches to more a comprehensive strategy, such as GWAS and whole genome sequencing, which will make it possible not only to identify novel genetic variants related to clinical outcomes, but also to analyze the effects of multiple genes on clinical outcomes. Extensive effort is required to apply pharmacogenetic information in clinical practice for a personalized medicine strategy of clozapine treatment.

## Author contributions

SN and HU selected the articles and wrote the first draft of the manuscript. RH and TO supervised and contributed to the editing the manuscript. All authors have approved the final manuscript.

### Conflict of interest statement

The authors declare that the research was conducted in the absence of any commercial or financial relationships that could be construed as a potential conflict of interest.
